# A review of breast cancer pathology reports in Nigeria

**DOI:** 10.3332/ecancer.2021.1190

**Published:** 2021-02-23

**Authors:** Adedayo O Joseph, Ya-Huei Li, Omolola Salako, Suhail Doi, Onyinye D Balogun, Opeyemi M Awofeso, Fatimah Abdulkareem, Adedayo A Onitilo

**Affiliations:** 1 NSIA-LUTH Cancer Treatment Center, Lagos University Teaching Hospital, Lagos, Nigeria; 2 Cancer Care and Research Center, Marshfield Clinic Research Institute, Marshfield, Wisconsin 54449, USA; 3 Department of Radiotherapy, College of Medicine, University of Lagos, Lagos, Nigeria; 4 Department of Population Medicine, College of Medicine, Doha, Qatar; 5 Department of Radiation Oncology, Weill Cornell Medicine, New York 10065, USA; 6 Lagos University Teaching Hospital, Lagos, Nigeria; 7 College of Medicine, University of Lagos, Lagos, Nigeria; 8 Department of Oncology, Marshfield Clinic Health System-Weston Center, 3501 Cranberry Blvd, Weston, WI 54476, USA; a https://orcid.org/0000-0001-9185-0606

**Keywords:** histopathologic report, pathology report completeness, biopsy and surgical specimens, Nigeria, resource-limited countries

## Abstract

**Background:**

Diagnosis and treatment of cancer rely heavily on imaging, histopathology and molecular information. Incomplete or missing tumour information can hinder the delivery of high-quality care in oncology practice, especially in resource-limited countries. To evaluate the completeness of histopathology reporting in a real-world setting and identify areas for future cancer care delivery research efforts, we retrospectively analysed reports from patients diagnosed with breast cancer who received care at a high-volume oncology department at a hospital in Lagos, Nigeria.

**Methods:**

Demographic, institutional and histopathology characteristics were retrospectively obtained from 1,001 patient records from 2007 to 2016. Completeness was defined as reporting five tumour features (tumour histology, tumour grade, laterality, oestrogen receptor (ER) or progesterone receptor (PR) and human epidermal growth factor receptor 2 (HER2)) for biopsy specimens and seven tumour features (tumour size, tumour histology, tumour grade, laterality, ER/PR, HER2 and lymph node involvement) for surgical specimens.

**Results:**

The mean age of patients was 48.6 ± 11.7 years with a predominantly female population (99.3%). A majority of pathologic reports were produced after 2011, and two-thirds of the reports originated from centres or labs within Lagos, Nigeria (67.7%). Most reports documented primary site (98.0%) and specimen type (85.0%) while other characteristics were less often recorded. This led to substantial variation in reporting between biopsy (13.4%) and surgical (6.1%) specimens for an overall low pathology report completeness <10%.

**Conclusion:**

The majority of patient records analysed lacked complete documentation of breast cancer histopathological characteristics commonly used in oncology practice. Our study highlights a need to identify and address the contributing factors for incomplete histopathological reporting in Nigeria and will guide future clinical programmatic developments.

## Introduction

Oncology treatment decisions are made based on information from clinical, radiologic and pathologic evaluation. Treatment decisions are heavily dependent on pathologic features that indicate or determine tumour aggressiveness, prognosis, treatment options, expected response to treatment and projected outcomes [1, 2]. In general, the management of breast cancer in Nigeria, like in other low- to middle-income countries, is wrought with many challenges. At initial presentation, advanced stage disease is usually seen, which can be attributed to lack of proper knowledge of the disease, socio-cultural and religious beliefs as well as a delay while seeking solutions from alternative care providers [3]. Following diagnosis, delays in initiation of definitive treatment, as a result of a patient’s low socioeconomic status or readiness and industrial action by tertiary health centres, often deter prompt management [4]. Furthermore, even when definitive treatment is initiated, there are attendant challenges to management including paucity of trained specialists, unavailability or unaffordability of radiotherapy services and low capacity for surgical intervention stemming from either late-stage presentation, fear of surgery or lack of access to quality surgical services [5, 6].

The delivery of individualised treatment in oncology is predicated upon accurate and adequate description of the biologic, cytologic and molecular description of the cancer, and it is difficult to develop an evidence-based individualised treatment plan with inadequate or inaccurate pathologic, molecular and cellular characterisation of the disease [7, 8]. In Nigeria, like many low- to middle-income countries, efforts to provide comprehensive pathology reporting remain incongruent and inadequate in many places [9–11]. The reasons for this are multifactorial and are undeniably impacted by socioeconomic and health-sector factors unique to the region [2, 12]. Some of these include lack of basic infrastructure like laboratory supplies, basic equipment and skilled personnel, as well as inadequate government policies on standardisation of laboratory testing, resulting in a lack of access to valuable pathological options including immunohistochemistry testing, and frozen sections that are valuable in the clinical outcome of breast cancer patients [13]. Some authors have documented improvement in various aspects of pathology reporting in the country, and there is a consensus to develop a uniformly accepted, implemented and monitored template for pathology reporting in the region [12, 14]. Several cross-sector national and global groups have proposed practice guidelines and developed a minimum set of reporting criteria to improve the standardisation of breast cancer pathology reporting worldwide [10]. Despite these efforts, there is still insufficient specimen documentation in Nigeria, with no nationally-enforced consensus on minimum reporting requirements. As such, generated reports can vary widely depending on the state, institution, centre or reporting laboratory, forcing treating oncologists to make decisions with inadequate data.

In 2015, the Pathology Department of the Lagos University Teaching Hospital (LUTH) proposed a standardised breast cancer reporting template for clinical practice [14]. This standardised template comprises eight essential groups of information to be included in the pathology report: organ site and type of operation, primary tumour diagnosis (tumour size, histological grade and extent of tumour), resection margins, lymph nodes, sentinel lymph nodes, other findings (lymphatic and/or vascular invasion) and ancillary studies (oestrogen receptor (ER), progesterone receptor (PR) and human epidermal growth factor receptor (HER2) expression) [14]. With the exception of an audit of histopathology reporting in patients with mastectomy to manage breast cancer in 2010 and a 2016 study to assess compliance with Royal College of Pathology guidelines in breast cancer reporting, there were few published studies evaluating the completeness of pathology reporting in Nigeria using global guidelines as a standard measure [15, 16].

The purpose of the current study was to explore the completeness of histopathology reports of patients diagnosed with invasive breast cancer seeking care at LUTH radiation oncology clinic and identify possible factors associated with incompleteness of histopathological reporting of breast cancer biopsies or surgical specimens to guide future research and programmatic efforts in Nigeria.

## Methods

### Institutional description

Serving as one of two tertiary centres in Lagos State, Nigeria, LUTH is a 761-bed hospital. It receives patients from the Lagos Metropolitan area, other states across the country and neighbouring African countries and treats over 4,000 patients with cancer annually [17]. Specifically, the oncology department of LUTH serves up to 500 patients with breast cancer referred to the centre by small hospitals and local clinics yearly [18]. Data collected from this hospital will provide relevant information regarding histopathological reporting in oncology practice in Nigeria.

### Patient selection

A total of 1,001 records of patients referred to the oncology department of LUTH for radio- or chemotherapy were randomly selected over a 10-year period from 2007 to 2016. Inclusion criteria included a diagnosis of breast cancer based on specimen site and diagnosis on record. Only the first visit associated with breast cancer treatment was considered if more than one visit was made. At the time of the study, recordkeeping was strictly manual, so data for this study was collected manually from paper charts and files. The laboratory from which the pathology reports originated had no digital registry as such, so paper printouts of the corresponding histopathology reports were reviewed, and data were extracted for analysis. Ethical approval was obtained from the hospital’s Health Research and Ethics Committee (Internal Review Board).

### Outcome of interest

The extent of histology report completeness prior to initiation of treatment at the oncology unit was the main outcome of interest. To account for differences in data quality among pathology reports originating from different sources, we extracted and evaluated data from pathology reports using different minimum requirements for specimen type. The definitions for completeness were set as follows for surgical and biopsy specimens. Five clinically relevant tumour features specifically tumour histology, tumour grade, laterality, ER/PR and HER2 completely documented for biopsy specimens, and seven tumour features specifically tumour size, tumour histology, tumour grade, laterality, ER/PR, HER2 and lymph node involvement completely documented for surgical specimens. Less than five tumour features documented in patients with biopsy specimens or less than seven tumour features documented in patients with surgical specimens were defined as incomplete; although, a few records had both surgical and biopsy specimen reports. The study focused on the analysis of the initial biopsy reports in all patients to evaluate for completeness.

### Data collection

Information extracted from the medical records and corresponding histology report of patients included individual characteristics such as sex, age and occupation, institutional factors like the year of report, reporting laboratory/centre (from Lagos versus other states, government versus private sector and teaching versus non-teaching institution) and histopathology characteristics as indicated above. Other information was also recorded such as the tumour features and type specimen including lumpectomies (referring to samples from excision biopsy), mastectomies or core needle biopsy. Lymph node involvement was defined as presence of or otherwise of lymph node status in the report. Where reported, total number of nodes excised or identified in the specimen as well as number of positive nodes was recorded. Pathology reports without full information regarding lymph node status—total number of nodes and number of nodes positive, were considered as incomplete. A few records had surgical and biopsy specimen reports; however, information was extracted only from the initial histopathological reports in all patients. These reports originated from several laboratories across the entire country, as a pathological report was mandatorily required for all cancer patients registering in the Oncology unit in LUTH. All data were recorded and stored in an electronic database.

### Statistical analysis

Descriptive statistics were reported for patient characteristics and tumour features, and chi-square test performed to analyse the statistical significance of the association between completeness (Yes/No) and categorical patient variables. The proportions of completeness were calculated as adding one more tumour feature up to a total of five or seven tumour features for biopsy or surgical specimens, respectively. Factors including the sociodemographic characteristics (age, sex, skillset), year of the report and the type of laboratory were compared with completeness of the pathological report to determine if a significant association was present. These factors are projected to influence the quality of the laboratory and possibly the quality and completeness of the reports generated. A *p*-value less than 0.05 was used as the threshold value for statistical significance. All statistical procedures were employed using SAS software (SAS Institute, Cary, NC, USA).

## Results

The descriptive reports of patient characteristics are presented in Table 1. The mean age of patients was 48.6 ± 11.7 years (range: 14–85 years old) with predominantly female patients (99.3%). A majority of pathology reports (83.5%) were produced after 2011, and two-thirds of the analysed reports originated from centres or laboratories within Lagos, Nigeria (67.7%). Many pathology reports were from private (55.2%) institutions or non-teaching hospitals (61.8%). Of the patients referred for breast cancer treatment, surgical and biopsy specimens accounted for 57.6% and 27.4% of pathology reports, respectively; while approximately 15% of patients did not have an associated pathology report or the source of pathology report was unknown. These patient records were grouped into the biopsy specimen subgroup because the majority of patients who had missing information in the specimen type were later confirmed to have a biopsy specimen after reaching out to the providers who referred the patient for clarification.

**Figure 1.**
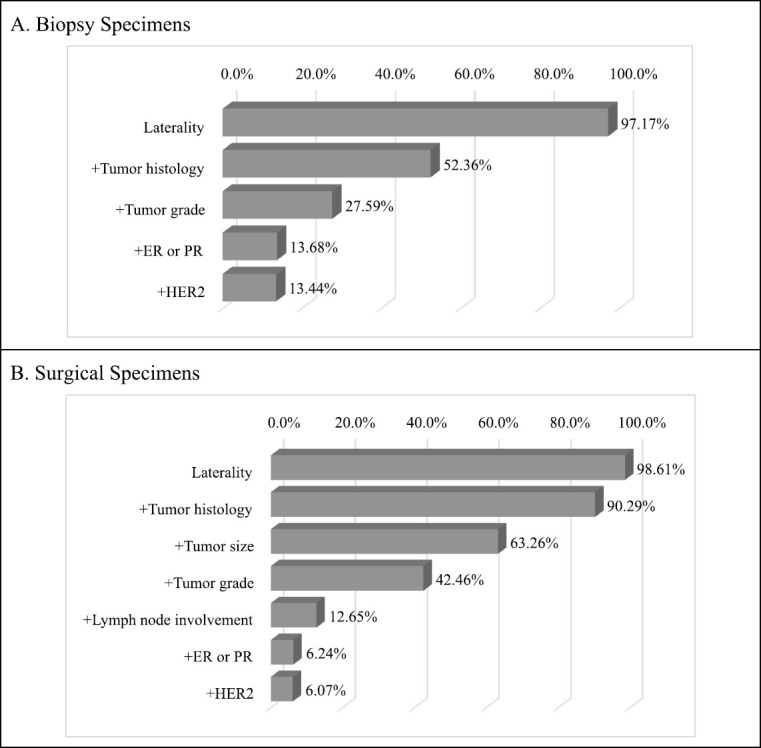
Identified completeness by specimen types in women with invasive breast cancer. Different tumour features were used for the evaluation of completeness of histopathology report in biopsy and surgical specimen subtypes. By iteratively adding tumour features, the percentage of completeness was changed. (a): Biopsy specimens: a total of five clinically relevant tumour features were considered for biopsy specimens: laterality, tumour histology, tumour grade, ER/PR and HER2. (b): Surgical specimens: a total of seven tumour features were considered for surgical specimens: laterality, tumour histology, tumour size, tumour grade, lymph node involvement, ER/PR and HER2.

Clinically relevant tumour features reported are summarised in Table 2. In a majority of the reports, tumour site (laterality: 98.0%) and specimen type (85.0%) were documented. However, reporting for other tumour characteristics ranged from 75.1% for tumour histology to almost 11% for lymphovascular space invasion. Analysis of documentation by specimen type revealed that documentation rates of the ten clinically essential tumour features were generally higher in surgical as compared to biopsy specimens. The overall rate of report completeness was less than 10%. Subgroup analysis of tumour characteristic by specimen type revealed additional significant variations in reporting of tumour grade, histology, margin and lymph node involvement (*p* < 0.0001) as well as lymphovascular space invasion (*p* = 0.0001) and ER or PR expression (*p* = 0.0181). Completeness rates of histopathology reporting by tumour characteristic are presented in Figure 1 according to specimen type. Patients with surgical specimens (90.3%) were more likely to have laterality and tumour histology reported compared to those with biopsy specimens (52.4%). In particular, the low reporting rate of lymph node involvement greatly reduced the completeness rate from 42.5% to 12.7% for women with surgical specimens. Furthermore, the total completeness rate of reports was lower in patients with surgical specimens (6.1%) than those with biopsy specimens (13.4%; *p* < 0.0001).

Table 3 highlights the potential factors associated with incomplete pathology reports. Variables of interest were patients’ ages, skill level, year of report, governmental sector reporting, type of laboratory (teaching versus non-teaching hospitals) and location of laboratory within or outside Lagos. Of these factors, only a more recent year of report (*p* = 0.0262) and being from hospitals/institutes within Lagos (*p* < 0.0005) were significantly associated with report completeness.

## Discussion

 The purpose of this study was to evaluate the completeness of breast cancer pathology reports received at a high-volume oncology department at a large hospital in Lagos, Nigeria. Although we noted improvements in histology reporting over time, our results indicated a continued incomplete reporting of specific breast tumour characteristics. This occurred even after publication of Mehta *et al*’s [1] proposed minimal requirement for breast specimen handling and reporting in 2010. Results indicate that only tumour laterality and specimen type were reliably and consistently documented in the referred patient reports; as such, if these were the minimum criteria by which completeness is defined, the rates would be significantly higher. In contrast, with the inclusion of the reporting rates of primary tumour diagnosis (tumour size, grade and histology), resection margin, lymphovascular space invasion, molecular biomarkers and pathological staging using the TNM staging system, the reports were relatively incomplete. Unsurprisingly, rates of reporting for ER, PR and human epidermal growth factor receptor-2 (HER2/neu) were the least recorded in this study, outlining the unavailability of immunohistochemistry in breast cancer management in Nigeria [19].

Since the diversity of clinical specimens for diagnosing cancer is so large, we focused our efforts on analysing the minimal requirements for surgical and biopsy specimens. However, with the proposed definitions of report completeness only focusing on these specimen types, the completeness of histopathology report rates was 13.4% and 6.1% for biopsy and surgical specimens, respectively. Despite the fact that surgical specimens are associated with higher report completion rates for ER/PR and HER2 expression than biopsy specimens [20], the completeness rate of this specimen type in the histopathology report overall is lower than biopsy specimens due to a higher number of tumour features analysed.

Compared to Daramola *et al*’s [16] evaluation of surgical specimen reports originating at a teaching hospital laboratory in Lagos, we noted a relatively lower reporting rate of tumour size (70.2% versus 100%), tumour grade (61.7% versus 89.6%), tumour margins (48.2% versus 50.4%) and sentinel lymph node (28.9% versus 40.0%). However, our report rates for tumour histology (91.2%) and immunohistochemistry (ER/PR: 33.6%, HER2: 31.7%) were higher (tumour histology: 27.8%, ER/PR/HER2: 26%). On the other hand, findings from another teaching hospital in Nigeria in 2010 showed lower report rates in tumour size (50% versus 70.2%), tumour grade (40% versus 61.7%) and lymphovascular space invasion (12% (arterial invasion emphasised) versus 14.2%) for patients with surgical specimens than those seen in our study [15]. These differences demonstrate that reporting practices from different pathology laboratories can vary widely.

Though report completeness differs across the region, some pathologists, in collaboration with international laboratories, have access to training, fellowships, remote interactions and observerships [9, 12]. These partnerships improve laboratory capacity, facilitate implementation and use of a standardised reporting system and connect pathologists to the most current information regarding histological techniques, laboratory guidelines and other continuing medical education activities [9, 21]. For those not fortunate to have such access, producing adequate histopathologic reports is challenging due to the scant number of both pathologists and laboratory support staff, competing demands on pathologist time (i.e. laboratory versus teaching duties), inadequate equipment and lack of access to continued training [2, 12]. Our results suggest that lack of access may be a contributing factor for missing histology report information, as we noted a significant difference in report completeness between pathology laboratories located within (12.1%) and outside of (4.6%) Lagos, Nigeria. Despite this discrepancy, overall completeness in histopathology reporting has not deteriorated over time, and in fact, we note a slight increase from 4.23% prior to 2010 to 12.24% in 2015–2016. Our study highlights variation in documentation of breast tumour characteristics in histopathology reports and reiterates a need for further investigations into the underlying factors associated with report incompleteness like laboratory location. Masood *et al.* [9] report that the challenges and barriers to developing quality histology reports in countries with limited resources are more difficult to conquer due to financial constraints, lack of trained pathologists and support staff, and inadequate or lack of equipment and reagents. With the definition of income classifications of the World Bank placing Nigeria in a similar category to India (i.e. a country with lower-middle income economy compared, for example, to Ethiopia, which is classified as having a low-income economy), identification and dissemination of health resources to areas of greatest need is essential to improving patient health [22]. Combined with preliminary analysis of histology reports in Pakistan and Ethiopia, our results suggest a possible association between the quality of histopathology reports and country income levels [10, 11]. However, it is important to note that financial and non-financial constraints at the patient level can also adversely impact the comprehensiveness of breast tumour analysis and other diagnostic tests [7, 23, 24]. Regardless of economics or cost, our results highlight incomplete pathology reporting of breast tumour characteristics that may impact treatment selection and ultimately patient care.

Though this study reiterated notable gaps in breast cancer pathology reports in Nigeria, our results need to be interpreted in light of the following limitations. Since we used a retrospective approach to evaluate the completeness of histopathology reports at admission over a 10-year period, histology reporting and documentation may have changed over time, and as such, the collected data may suffer from incomplete or biased documentation. For example, we note that approximately 15% of patient records had either missing pathology information, or the pathology report source was unknown or undocumented. To account for this inherent limitation in study design, we stratified the cohort into three periods: prior to 2010, 2011–2015 and 2015–2016. This stratification was performed to account for implementation of reporting changes at LUTH in 2015. As we expected, the percentage of report completeness improved over time. A second study limitation is that only a few tumour features were considered for evaluation of completeness in histopathology reports, though more elements are critically needed for decision-making in care. Third, only the completeness of the actual report was evaluated; we did not study testing accuracy or the information transfer processes between institutions that may help or hinder reporting for associated patient records. Reasons for missing pathology information may be a failure on the part of the physician to order a laboratory test such as immunohistochemistry, inappropriate specimen collection and processing prior to pathology analysis, incomplete report transfer to the laboratory or referring facility or inadequate tissue handling and processing in the laboratory [9–12, 23, 25]. Finally, only two types of samples were reviewed for analysis from patients referred to a single clinical oncology department at a large academic medical centre. Information generated from this study may not be completely generalisable for other specimen types or hospitals in Nigeria. However, as we evaluated more than a thousand cases and approximately 100 invasive breast cancers (20%) annually between 2011 and 2016 for a large tertiary centre that receives a large percentage of individuals from Nigeria and surrounding countries, we consider this generalisable to the Nigerian context.

Despite these limitations, our study offers some insight into the real-world experience of clinical oncologists at an academic medical centre in a lower-middle income nation. Although reporting of more disease-related features is desirable for treatment decision-making, it is essential to acknowledge and identify the multifactorial challenges and barriers to achieving high quality histopathology reports in low-resource settings [25]. We support a multidimensional approach including national awareness and patient advocacy for cancer care, funding of health care education and facilities, surgical care and standardisation, and increased pathologist and surgeon access to continuing medical education activities to enhance histopathology reporting practice in Nigeria.

## Conclusion

Our results reveal a continuing incompleteness of reporting of breast cancer tumour characteristics in Nigeria and support the need for future research efforts to identify and mitigate factors that adversely impact histopathology reporting across the country. Furthermore, we encourage the increased establishment of institutional, laboratory and hospital collaborations between urban and rural areas to promote resource and health information sharing across the country.

## List of abbreviations

ER, Oestrogen receptor; PR, Progesterone receptor; HER2, Human epidermal growth factor receptor 2; LUTH, Lagos University Teaching Hospital

## Research presentations

This work was presented in part at the 2018 UICC World Cancer congress Malaysia.

## Conflicts of interest

There are no conflicts of interest to report on the part of any of the authors.

## Funding statement

The authors received no financial support for this research.

## Figures and Tables

**Table 1.** Patient record characteristics (*N* = 1,001).

Patient record characteristics	*N* (%)
**Age**	
Mean ± SD; Range (years old)	48.6 ± 11.7; 14–85
≤40	265 (26.82)
41–50	327 (33.10)
51–60	234 (23.68)
≥61	162 (16.40)
**Sex**	
Female	994 (99.30)
Male	7 (0.70)
**Skill level^a^**	
Professional	283 (28.27)
Semi-skilled	70 (6.99)
Unskilled	492 (49.15)
Retired	71 (7.09)
Not applicable	85 (8.49)
**Year of report**	
Prior 2010	142 (16.53)
2011–14	333 (38.77)
2015–16	384 (44.70)
**Report from Lagos**	
No	280 (32.30)
Yes	587 (67.70)
**Report produced by government sector**	
No	478 (55.20)
Yes	388 (44.80)
**Report produced by the laboratory at a teaching hospital**	
No	534 (61.81)
Yes	330 (38.19)
**Specimen types**	
Lumpectomy	156 (15.58)
Mastectomy	419 (41.86)
Quadrant	2 (0.20)
Core needle biopsy	151 (15.08)
Fine needle aspiration cytology	123 (12.29)
No record	150 (14.99)

a Professional: Teachers, civil servants, nurses, engineers, accounts, bankers, doctors and others.

Semi-skilled: Fish farmers, fashion designers, distributors, caterers, hair stylists, administrative officers and others.

Unskilled: Housewives, traders, contract staffs, traders, clergies and others

**Table 2.** Clinically significant tumour features reported in the study cohort.

Tumour features	All sample *N* (%)	Biopsy^a^ *N* (%)	Surgical *N* (%)	*p* value
Specimen type	851 (85.01)	424	577	
Laterality	981 (98.00)	412 (97.17)	569 (98.61)	0.1068
Tumour size	695 (69.43)	290 (68.40)	405 (70.19)	0.5426
Tumour grade	488 (48.75)	132 (31.13)	356 (61.70)	<0.0001
Tumour histology	752 (75.12)	226 (53.30)	526 (91.16)	<0.0001
Tumour margin	347 (34.67)	69 (16.27)	278 (48.18)	<0.0001
Lymph node involvement	185 (18.48)	18 (4.25)	167 (28.94)	<0.0001
Lymphovascular space invasion	110 (10.99)	28 (6.60)	82 (14.21)	0.0001
ER or PR	307 (30.67)	113 (26.65)	194 (33.62)	0.0181
HER2	294 (29.37)	111 (26.18)	183 (31.72)	0.0574
Scarff–Bloom–Richardson histologic grading	260 (25.97)	86 (20.28)	174 (30.16)	0.0004
Completeness of tests/examinations^b^	92 (9.19)	57 (13.44)	35 (6.07)	<0.0001

ER, Oestrogen receptor; PR, progesterone receptor; HER2, human epidermal growth factor receptor 2**^a^**Biopsy specimen subgroup comprises both biopsy and no record in the specimen type^b^*Completeness* is defined as a pathology report including five clinically relevant tumour features (tumour histology, tumour grade, laterality, ER/PR and HER2) completely documented for a biopsy specimen or seven tumour features (tumour size, tumour histology, tumour grade, laterality, ER/PR, HER2 and lymph node involvement) completely documented for a surgical specimen. *Incompleteness* is defined as report missing one or more tumour features for the pre-specified sample types

**Table 3.** Association between variables of interest and completeness of reporting (chi-squared test).

Variables of interest	Complete *N* (%)	Incomplete *N* (%)	*p* value
**Age category**			0.3185
≤40	18 (6.79)	247 (93.21)	
41–50	30 (9.17)	297 (90.83)	
51–60	26 (11.11)	208 (88.89)	
≥61	18 (11.11)	144 (88.89)	
**Sex**			0.6396
Female	91 (9.15)	903 (90.85)	
Male	1 (14.29)	6 (85.71)	
**Skill level**			0.6044
Professional	30 (10.6)	253 (89.4)	
Semi-skilled	5 (7.14)	65 (92.86)	
Unskilled	40 (8.13)	452 (91.87)	
Retired	9 (12.68)	62 (87.32)	
Not applicable	8 (9.41)	77 (90.59)	
**Year of report**			0.0262
Prior 2010	6 (4.23)	136 (95.77)	
2011–14	35 (10.51)	298 (89.49)	
2015–16	47 (12.24)	337 (87.76)	
**Report from Lagos**			0.0005
No	13 (4.64)	267 (95.36)	
Yes	71 (12.10)	516 (87.90)	
**Report produced by government sector**			0.5429
No	49 (10.25)	429 (89.75)	
Yes	35 (9.02)	353 (90.98)	
**Report produced by the laboratory at a teaching hospital**			0.4662
No	55 (10.30)	479 (89.70)	
Yes	29 (8.79)	301 (91.21)	

*Completeness* is defined as a pathology report including five clinically relevant tumour features (tumour histology, tumour grade, laterality, ER/PR and HER2) completely documented for a biopsy specimen or seven tumour features (tumour size, tumour histology, tumour grade, laterality, ER/PR, HER2 and lymph node involvement) completely documented for a surgical specimen. *Incompleteness* is defined as report missing one or more tumour features for the pre-specified sample typesSample size: Complete (*N* = 92), Incomplete (*N* = 909)
